# Piezocatalytically enhanced antibacterial and antibiofilm performance of biogenic vanadium nanoparticles from *Pasteurella aerogenes*

**DOI:** 10.1039/d5na01132a

**Published:** 2026-07-07

**Authors:** Karzan Qurbani, Safin Hussein, Bahra Abbas Ibrahim, Hedi Muhammadamin Khalid, Doaa K. Al-seleet, Abdulmalik Fareeq Saber, Vania Hassan Muhammed, Shnan Abdulrahman Hassan, Saman M. Mohammed

**Affiliations:** a Biology Department, College of Science, University of Raparin Ranya Sulaimaniya Kurdistan Region Iraq safin.hussein@uor.edu.krd; b Adult Nursing Department, College of Nursing, University of Sumer Dhi Qar Iraq; c Nursing Department, Lebanese French University Erbil Kurdistan Region Iraq; d Department of Biology, College of Education, University of Sulaimani Sulaymaniyah Kurdistan Region Iraq

## Abstract

Antimicrobial resistance (AMR) and environmental pollution are escalating global threats necessitating the development of sustainable, multifunctional nanomaterials. This study reports, to the best of our knowledge, the first eco-friendly biosynthesis of vanadium nanoparticles (VNPs) using the metal-tolerant bacterium *Pasteurella aerogenes*. The bacterium demonstrated remarkable tolerance to vanadium (up to 20 mM), facilitating the enzymatic reduction and stabilization of predominantly spherical, crystalline VNPs (20–80 nm), confirmed by FESEM, TEM, EDX, DLS, and XRD. Optimization studies identified ethanol-mediated lysis followed by calcination at 250 °C as the ideal synthesis condition. The VNPs exhibited high piezocatalytic activity, achieving 88.0% methylene blue degradation under ultrasonic activation, primarily driven by hydroxyl radicals (˙OH), with pseudo-first-order kinetics confirmed at the optimal temperature (60 °C, *k*_app_ = 0.1933 min^−1^). The enhanced activity under ultrasonication is consistent with acoustic-driven piezocatalytic mechanisms supported by published evidence for vanadium oxide piezoelectric behavior. Furthermore, ultrasonicated VNPs demonstrated potent concentration-dependent antibacterial and antibiofilm efficacy against multidrug-resistant *Staphylococcus aureus* and extensively drug-resistant *Pseudomonas aeruginosa*, achieving >95% biofilm inhibition at 125 µg mL^−1^ for both pathogens. These findings establish biogenic VNPs as a promising nanoplatform for pollutant remediation and AMR mitigation, pending further *in vivo* validation.

## Abstract 2

Eco-friendly vanadium nanoparticles synthesized by *Pasteurella aerogenes* exhibit potent piezocatalytic pollutant degradation and antibacterial/antibiofilm activity against drug-resistant pathogens under ultrasonic stimulation.

## Graphical abstract

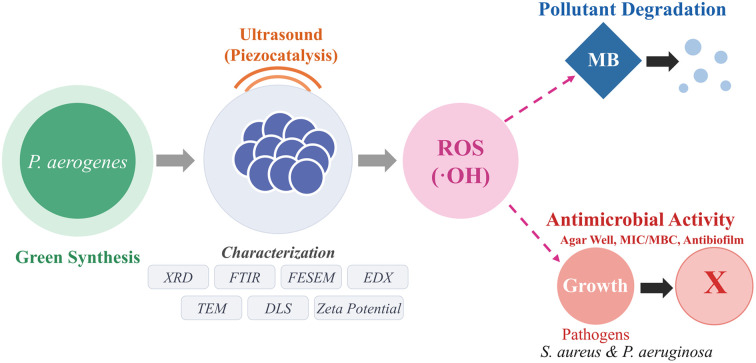

## 1. Introduction

Antimicrobial resistance (AMR) represents one of the most critical threats to global public health, environmental sustainability, and modern medicine. Pathogenic bacteria, including *Staphylococcus aureus* and *Pseudomonas aeruginosa*, have developed multidrug-resistant (MDR) and extensively drug-resistant (XDR) phenotypes that severely compromise conventional antibiotic efficacy.^1^ The rapid dissemination of resistance mechanisms has outpaced the development of new therapeutic agents. This dissemination is driven by antibiotic overuse, inadequate infection control practices, and horizontal gene transfer. Modeling studies project that in the absence of innovative interventions, AMR-related deaths could escalate to ten million annually by 2050.^2^ This alarming scenario necessitates the development of alternative antimicrobial strategies that operate through mechanisms distinct from traditional antibiotics, thereby circumventing existing resistance pathways.

Nanotechnology has emerged as a transformative approach to combat AMR through the design of materials with unique physicochemical properties at the nanoscale (1–100 nm).^3^ Nanoparticles exhibit enhanced surface reactivity, quantum effects, and high surface-area-to-volume ratios that enable potent antimicrobial activity through multiple mechanisms including membrane disruption, protein denaturation, and reactive oxygen species (ROS) generation.^4^ These multifaceted modes of action reduce the likelihood of resistance development compared to single-target antibiotics. However, conventional chemical and physical synthesis methods present significant limitations. Chemical approaches often require toxic reducing agents, organic solvents, and harsh reaction conditions that generate hazardous byproducts and limit biomedical applications.^5^ Physical methods, while precise, are energy-intensive and economically prohibitive for large-scale production.^6^

These constraints have catalyzed interest in green synthesis methodologies that employ biological systems as eco-friendly alternatives. Biosynthesized nanoparticles demonstrate superior biocompatibility, stability, and environmental safety while operating under ambient conditions.^7^ Biological synthesis harnesses the reducing capacity of natural systems—including plants, fungi, algae, and bacteria—to convert metal precursors into stable nanostructures without toxic intermediates.^8,9^

Bacteria are particularly promising nanofactories due to their rapid growth rates, metabolic diversity, and enzymatic machinery capable of metal ion reduction.^10^ Bacterial extracellular synthesis facilitates straightforward nanoparticle recovery and offers scalability advantages over intracellular approaches. While biosynthesis of silver, gold, and zinc oxide nanoparticles has been extensively documented, vanadium nanoparticles (VNPs) remain significantly underexplored despite their distinctive properties.^11,12^ Vanadium's multiple oxidation states (V^2+^ to V^5+^) enable efficient redox cycling and catalytic activity, generating oxidative stress in microbial cells through sustained ROS production.^13^ These characteristics position vanadium-based nanomaterials as promising candidates for antimicrobial applications.

Recent advances in nanomaterial science have revealed that certain semiconducting nanoparticles exhibit piezocatalytic properties, which refers to the ability to generate electric charges and reactive species under mechanical stress or ultrasonic stimulation.^14^ When subjected to mechanical deformation, piezoelectric materials develop internal electric fields that drive redox reactions at their surfaces, producing hydroxyl radicals (˙OH) and superoxide anions (O_2_˙^−^) without external chemical oxidants or light irradiation.^15^ This piezocatalytic mechanism offers distinct advantages for antimicrobial applications: it can be activated by ultrasonic waves or physiological vibrations, enables deep-tissue treatment, and provides on-demand ROS generation that minimizes off-target toxicity.^16^ Recent studies have demonstrated that vanadium dioxide exhibits piezoelectric properties under strain-induced structural modifications, with piezoelectric coefficients comparable to conventional piezoelectric materials, making it a promising candidate for piezocatalytic antibacterial applications.^17^ However, the piezocatalytic potential of biogenically synthesized VNPs remains unexplored, representing a significant knowledge gap in the integration of green synthesis with advanced catalytic antimicrobial mechanisms.

*Pasteurella aerogenes* represents an ideal biological platform for VNP synthesis due to its metabolic versatility and metal-stress tolerance. This facultative anaerobe secretes extracellular oxidoreductases and dehydrogenases that facilitate electron transfer from cellular metabolites to metal ions, enabling controlled nanoparticle formation.^18^ The extracellular synthesis pathway simplifies downstream processing and enhances scalability. Furthermore, the biological capping agents produced during microbial synthesis may influence the piezoelectric properties and surface reactivity of the resulting nanoparticles, potentially enhancing their antimicrobial efficacy through synergistic mechanisms.

Despite growing interest in both green nanosynthesis and piezocatalytic materials, critical knowledge gaps remain. While vanadium oxide nanomaterials^19^ and piezocatalytic vanadate systems^20^ have been previously reported, including the biogenic fabrication of vanadium dioxide using strains like *Shewanella* sp.,^21^ the biogenic synthesis of VNPs using *P. aerogenes* specifically has not, to our knowledge, been previously described. Nor has the piezocatalytic enhancement of antimicrobial activity been examined in biogenically synthesized VNPs. Additionally, the mechanisms underlying biofilm disruption by piezocatalytically activated nanomaterials against MDR and XDR pathogens remain poorly understood. Biofilm-associated infections are particularly recalcitrant to treatment, as the extracellular polymeric matrix protects embedded bacteria from antimicrobial agents and immune responses.^22^

This study addresses these gaps by developing an eco-friendly biosynthesis protocol for VNPs using *P. aerogenes* and evaluating their piezocatalytically enhanced antibacterial and antibiofilm activity against clinical isolates of MDR *S. aureus* and XDR *P. aeruginosa*. The specific objectives are to: (i) optimize biosynthesis parameters to achieve controlled nanoparticle formation with desirable physicochemical properties; (ii) comprehensively characterize the structural, morphological, and piezoelectric properties of the biosynthesized VNPs; (iii) assess their antibacterial efficacy in planktonic and biofilm modes under both static and ultrasonic conditions; and (iv) elucidate the mechanisms of action, with emphasis on piezocatalytic ROS generation and membrane damage. The findings will advance sustainable nanotechnology, provide mechanistic insights into piezocatalytic antimicrobial systems, and offer a novel therapeutic platform applicable to biomedical devices, wound care, pharmaceutical formulations, and environmental remediation. This research aligns with global initiatives to develop environmentally conscious technologies that address AMR through biologically inspired nanoscience and advanced catalytic mechanisms.

## 2. Materials and methods

### 2.1 Bacterial strain and vanadium tolerance

*P. aerogenes*, previously isolated from heavy-metal-contaminated soil and molecularly identified through 16S rRNA sequencing,^18^ was employed as the biological agent for vanadium nanoparticle (VNP) biosynthesis. The bacterium's vanadium tolerance was determined using a broth microdilution assay performed in sterile 96-well microplates.^23^ Ammonium metavanadate (NH_4_VO_3_) was prepared in Luria–Bertani (LB) broth (pH 7.0) at concentrations ranging from 0 to 20 mM. Each well received 180 µL of LB medium and 20 µL of bacterial inoculum standardized to 1.0 × 10^8^ CFU mL^−1^. Plates were incubated at 30 °C under orbital shaking (180 rpm) for 24 h, and bacterial growth was quantified spectrophotometrically at optical density readings at 600 nm (OD_600_) using a microplate reader (ELx808, BioTek). To determine the maximum tolerable concentration (MTC), 5 µL aliquots from wells showing no visible turbidity were spot-plated onto nutrient agar and incubated at 30 °C for 24 h.^24^

### 2.2 Characterization of synthesized nanoparticles

The physicochemical properties of the biosynthesized VNPs were characterized using X-ray diffraction (XRD), Fourier-transform infrared spectroscopy (FTIR), scanning electron microscopy (SEM), energy-dispersive X-ray spectroscopy (EDX), transmission electron microscopy (TEM), dynamic light scattering (DLS), and zeta potential analysis. XRD patterns were obtained using a Philips X'Pert PRO diffractometer equipped with Cu Kα radiation (*λ* = 1.5406 Å), operated at 40 kV and 30 mA, over a 2*θ* range of 10°–80° with a scanning rate of 0.02° s^−1^. FTIR spectra were recorded on a PerkinElmer Spectrum Two spectrometer within the 4000–400 cm^−1^ region to identify surface functional groups responsible for the reduction and stabilization of vanadium ions.

Morphological characteristics and surface topology were examined by field emission SEM (FESEM; MIRA3, TESCAN, Czech Republic) operated at an accelerating voltage of 15.0 kV at a magnification of 135k×; samples were deposited onto aluminum stubs and sputter-coated with gold prior to imaging. Elemental composition was confirmed by energy-dispersive X-ray spectroscopy (EDX) performed on the same instrument, with elemental mapping acquired to assess the spatial distribution of constituent elements. TEM analysis was performed to confirm particle morphology and size in the non-aggregated state; samples were prepared by depositing a droplet of dilute VNP suspension onto carbon-coated copper grids followed by complete air-drying prior to imaging. Hydrodynamic particle size, PDI, and zeta potential were determined by DLS using a HORIBA SZ-100 nanoparticle analyzer (HORIBA Scientific, Japan) at a scattering angle of 90° and a temperature of 24.7–24.8 °C, with a dispersion medium viscosity of 0.900–0.903 mPa s; all measurements were performed in triplicate (*n* = 3).

### 2.3 Optimization of biosynthesis and post-processing conditions

A mid-logarithmic-phase culture of *P. aerogenes* (OD_600_ = 0.8) was cultivated in LB medium at 30 °C with shaking (150 rpm). Cells were harvested by centrifugation (6000*g*, 10 min), washed twice with ultrapure water, and resuspended to an OD_600_ = 1.0. For nanoparticle synthesis, the bacterial biomass was exposed to 5 mM NH_4_VO_3_ in LB medium and incubated at 30 °C under continuous agitation (150 rpm).^25^ To determine optimal biosynthetic conditions, the incubation period, cell-disruption technique, and calcination temperature were systematically varied (Table 1). Incubation was performed for 24, 48, 72, and 96 h to assess the influence of exposure time on metal-ion bioreduction efficiency and nanoparticle yield.

**Table 1:** Experimental conditions used for the biosynthesis and optimization of vanadium nanoparticles

Sample code	Incubation time (h)	Cell-disruption method	Calcination temperature (°C)
V1	24	Autoclave (121 °C, 15 psi, 20 min)	250
V2	48	Autoclave (121 °C, 15 psi, 20 min)	250
V3	72	Autoclave (121 °C, 15 psi, 20 min)	250
V4	96	Autoclave (121 °C, 15 psi, 20 min)	250
V5	48	Autoclave (121 °C, 15 psi, 20 min)	150
V6	48	Autoclave (121 °C, 15 psi, 20 min)	250
V7	48	Ethanol lysis (70% v/v, 22 °C, 20 min)	150
V8	48	Ethanol lysis (70% v/v, 22 °C, 20 min)	250

Intracellularly formed VNPs were recovered using two different disruption strategies: (i) thermal treatment at 121 °C and 15 psi for 20 min, and (ii) ethanol-mediated lysis using 70% (v/v) ethanol for 20 min at 22 °C.^26^ The resulting lysates were centrifuged (15 000*g*, 20 min), and the pellets containing crude VNPs were subjected to calcination at 0, 150, and 250 °C for 160 min to improve crystallinity and phase purity.^27^ Following calcination, VNPs were redispersed in analytical-grade ethanol, purified by high-speed centrifugation (14 000 rpm, 30 min), and stored at 4 °C for further characterization and antibacterial evaluation.

### 2.4 Catalytic and piezocatalytic activity assessment

The catalytic efficiency of the biosynthesized VNPs was evaluated and optimized through systematic investigation of methylene blue (MB) dye degradation as a model reaction. Ultrasonicated VNP suspensions (300 W) were prepared at a concentration of 100 µg mL^−1^ and mixed with an MB solution (5 µg mL^−1^). The degradation kinetics were monitored spectrophotometrically at *λ*_max_ = 664 nm using a UV-vis spectrophotometer.

To optimize catalytic performance, VNP suspensions were subjected to varying ultrasonication durations (0–60 min) and temperatures (20, 40, 60 °C) under a constant power of 300 W. Each sonication time was examined at three different temperatures (20, 40, and 60 °C) to systematically determine the optimal combination of duration and temperature that yielded the highest catalytic activity. After each treatment, the reaction mixtures were centrifuged (10 000*g*, 10 min), and the supernatants were analyzed to quantify residual MB concentration.

The degradation kinetics were subsequently evaluated by fitting the experimental data to zero-order, pseudo-first-order, and second-order kinetic models. Owing to the proportional relationship between absorbance and concentration described by the Beer–Lambert law, absorbance values were used to estimate the corresponding MB concentrations. The apparent rate constants and coefficients of determination (*R*^2^) were obtained by linear regression analysis to identify the kinetic model that best described the degradation process.

The piezocatalytic performance of the optimized VNPs was evaluated under different mechanical activation conditions. Additional treatments comprised low-to-moderate shear generated by mechanical stirring at 100 and 250 rpm, high shear at 1000 rpm, and ultrasonication (300 W, 5 min). Two controls were included: a VNPs (0 rpm) control (VNPs + MB without mechanical activation) to quantify passive dye adsorption onto the VNP surface in the absence of piezocatalytic activation, and an MB + US control (MB subjected to ultrasonication at 300 W for 5 min without VNPs) to evaluate the contribution of acoustic cavitation alone to MB removal.

Reaction systems containing 100 µg mL^−1^ VNPs and 5 µg mL^−1^ MB solution were exposed to these mechanical regimes at 40 ± 2 °C. After treatment, the suspensions were centrifuged, and the absorbance of the residual MB at 664 nm was recorded.

Catalytic degradation efficiency (*η*) was calculated using the equation:
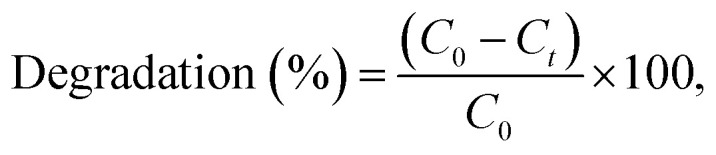
where *C*_0_ and *C*_*t*_ denote the initial and final absorbance values of MB, respectively. The optimized sonication time and temperature identified from these experiments were subsequently applied in the mechanistic and antibacterial investigations.

### 2.5 Reactive species investigation and mechanisms of action

The generation of ROS during piezocatalytic activity of the biosynthesized VNPs was investigated using radical scavenging assays to identify the predominant reactive species. Specific scavengers were employed: benzoquinone (BQ) for ˙O_2_^−^, isopropanol (IP) for ˙OH, and methanol for photogenerated valence-band holes (h^+^). Each reaction system contained 5 ppm of MB, and VNPs (100 µg mL^−1^), with untreated controls serving to establish baseline degradation efficiency.

All experimental setups were subjected to ultrasonication at 300 W for 5 min to activate piezocatalysis. Following treatment, the suspensions were centrifuged, and the residual concentration of MB was quantified spectrophotometrically at *λ*_max_ = 664 nm using a UV-vis spectrophotometer (Shimadzu UV-1800). The degradation efficiency (*η*) was calculated according to the following equation:
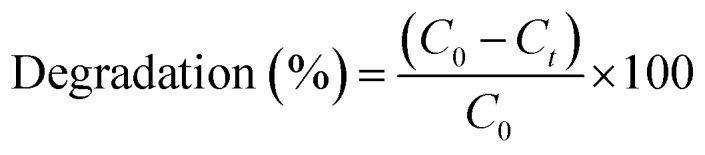
where *C*_0_ and *C*_*t*_ represent the initial and final absorbance values of MB, respectively. A decrease in degradation efficiency in the presence of a given scavenger was interpreted as indicative of the corresponding ROS being actively involved in the piezocatalytic mechanism.

### 2.6 Antimicrobial activity evaluation

#### 2.6.1 Agar well diffusion assay

The antibacterial activity of the biosynthesized VNPs was evaluated using the agar well diffusion method against MDR *S. aureus* and XDR *P. aeruginosa*. The isolates were obtained from routine diagnostic specimens processed at Shar Hospital, Sulaymaniyah, Kurdistan Region of Iraq. Species identification and antimicrobial susceptibility testing were performed using the BD Phoenix™ automated microbiology system in accordance with Clinical and Laboratory Standards Institute (CLSI) interpretive guidelines. MDR and XDR classifications were assigned based on resistance to multiple antimicrobial classes according to internationally accepted consensus definitions. Detailed characterization and antimicrobial susceptibility profiles have been reported previously.^28^ Standardized bacterial suspensions (0.5 McFarland, approximately 1.5 × 10^8^ CFU mL^−1^) were inoculated onto sterile Mueller–Hinton agar (MHA) plates to obtain uniform lawn cultures. Wells of 6 mm diameter were aseptically created in the agar and filled with 100 µL of VNP suspension at a concentration of 500 µg mL^−1^. VNPs were subjected to ultrasonication (300 W, 5 min) prior to addition to bacterial cultures; bacteria were not directly exposed to ultrasonication at any point during the assay. This approach was applied consistently across all antibacterial and antibiofilm assays (Sections 2.6.2 and 2.6.3).

To assess the effect of piezocatalytic activation, two parallel tests were conducted: one using VNPs without sonication and the other using VNPs after ultrasonication at 300 W for 5 min. The plates were incubated at 37 °C for 24 h, and the diameters of the inhibition zones were measured in millimeters (mm). All experiments were performed with multiple replicates to ensure reproducibility. Azithromycin (15 µg disc) was used as the standard antibiotic for comparison with the antibacterial activity of the synthesized VNPs.

#### 2.6.2 MIC and MBC determination

The minimum inhibitory concentration (MIC) and minimum bactericidal concentration (MBC) of the sonicated VNPs (VNPs; 300 W, 5 min) were determined against MDR *S. aureus* and XDR *P. aeruginosa* using the broth-microdilution technique. Serial VNP concentrations (0, 25, 50, 75, 100, 125, 250, and 500 µg mL^−1^) were prepared in sterile Mueller–Hinton broth.

Sterile 96-well microplates were inoculated with 10 µL of standardized bacterial suspension (0.5 McFarland standard, approximately 1.5 × 10^8^ CFU mL^−1^), 25 µL of the sonicated VNP suspension, and Mueller–Hinton broth to a final volume of 200 µL per well. Plates were incubated at 37 °C for 24 h, and bacterial growth was quantified spectrophotometrically at OD_600_ using a microplate reader (BioTek ELx808).

The MIC was defined as the lowest VNP concentration that completely inhibited visible bacterial growth. To determine the MBC, 5 µL aliquots from wells showing no visible turbidity were spot-plated onto nutrient agar and incubated at 37 °C for 24 h. The MBC was recorded as the lowest nanoparticle concentration producing no bacterial colonies, confirming complete bactericidal activity.

#### 2.6.3 Antibiofilm activity assessment

The antibiofilm efficacy of the sonicated VNPs (300 W, 5 min) was evaluated using the crystal-violet microtiter-plate method. Serial concentrations of VNPs (0, 25, 50, 75, 100, 125, 250, and 500 µg mL^−1^) were tested against MDR *S. aureus* and XDR *P. aeruginosa*. Standardized bacterial suspensions (0.5 McFarland, approximately 1.5 × 10^8^ CFU mL^−1^) were inoculated into sterile 96-well microplates and incubated statically at 37 °C for 48 h to allow biofilm formation.

Following incubation, non-adherent cells were gently removed, and the wells were washed three times with sterile phosphate-buffered saline (PBS, pH 7.4). The adherent biofilms were stained with 0.1% (w/v) crystal violet for 15 min, rinsed to remove excess dye, and the retained stain was solubilized with 200 µL of 96% ethanol. Absorbance was measured at 570 nm using a microplate reader (BioTek ELx808).

The percentage of biofilm inhibition was calculated as:
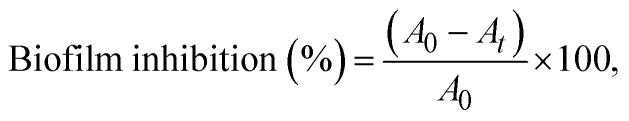
where *A*_0_ represents the absorbance of the untreated control and *A*_*t*_ the absorbance of VNP-treated wells. All assays were performed in triplicate, and results were expressed as mean ± standard deviation.

### 2.7 Statistical analysis

All experiments were performed in triplicate (*n* = 3) and results are presented as mean ± standard deviation. One-way ANOVA with Tukey's or Dunnett's *post hoc* test was used for single-factor comparisons, and two-way ANOVA with Tukey's or Dunnett's *post hoc* test for two-factor comparisons, with *p* < 0.05 considered statistically significant. All analyses and graphs were generated using GraphPad Prism software.

## 3. Results and discussion

### 3.1 Bacterial tolerance and nanoparticle biosynthesis

**Fig. 1:**
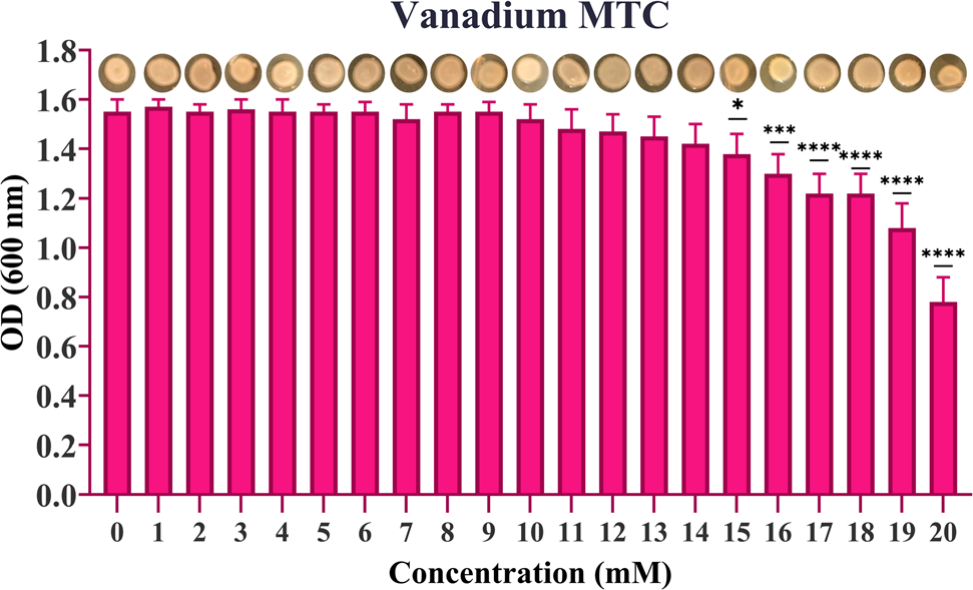
Determination of the maximum tolerable concentration (MTC) of vanadium for *P. aerogenes*. Bacterial growth (OD_600_) across vanadium concentrations (0–20 mM) shows a significant decline beginning at 15 mM (one-way ANOVA with Dunnett's *post hoc* test *vs.* 0 mM control; *p < 0.05, ***p < 0.001, ****p < 0.0001; *n* = 3, error bars represent SD). Insets: corresponding spot assays of *P. aerogenes* on nutrient agar at each tested concentration.

The growth of *P. aerogenes* in liquid cultures containing VNPs was monitored through OD_600_ (Fig. 1). From 0 to 10 mM, OD_600_ values remained stable between 1.5 and 1.62. A gradual decline in growth became evident starting at 11 mM, with OD_600_ values decreasing to approximately 1.4 at 14 mM and reaching approximately 0.8 at 20 mM. Greater variability in bacterial response was observed at higher concentrations (16–20 mM). However, complete growth inhibition was not observed at any tested concentration, and no definitive MTC was identified within the tested range of 0–20 mM. Spot assays on nutrient agar plates (Fig. 1, insets) showed visible bacterial colonies across all vanadium concentrations tested. Colony density and morphology remained consistent from 0 to 14 mM, with slight reductions visible at concentrations above 14 mM.

This study established *P. aerogenes* as a robust biological platform for the eco-friendly synthesis of VNPs. The pronounced tolerance of *P. aerogenes* to vanadium concentrations up to 20 mM highlights its exceptional adaptability to metal-induced stress. This high tolerance likely reflects multiple protective mechanisms, including the secretion of extracellular polymeric substances, production of metal-chelating enzymes, and expression of oxidoreductases involved in detoxification.^29^ Comparable mechanisms have been reported in *Pseudomonas* species, where metal ion transporters and efflux pumps maintain intracellular homeostasis under heavy-metal exposure.^30,31^ Such metal resistance establishes *P. aerogenes* as a promising platform for sustainable biosynthesis of transition-metal nanoparticles.

### 3.2 Physicochemical characterization of VNPs

**Fig. 2:**
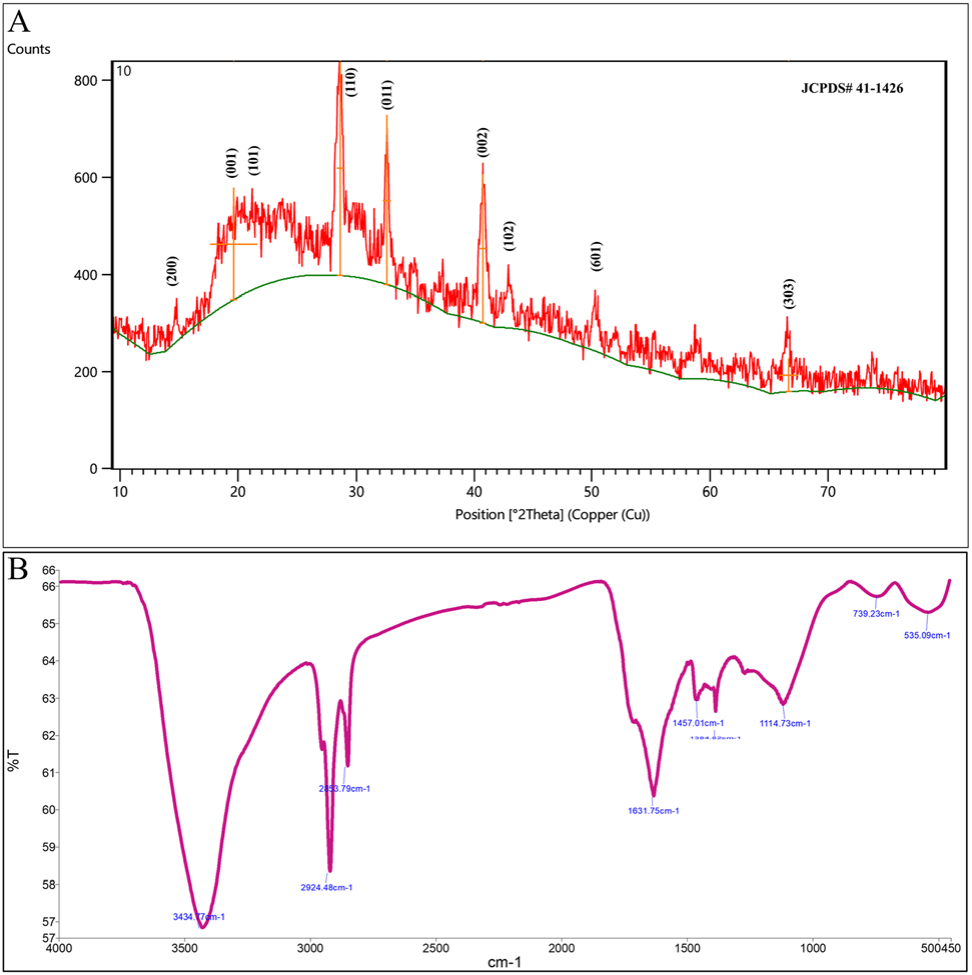
Characterization of VNPs synthesized from *P. aerogenes* extracts. (A) XRD pattern of synthesized VNPs (red line) with background trend (green line), showing diffraction peaks indexed to V_2_O_5_ (JCPDS no. 41-1426) with corresponding Miller indices. (B) FTIR spectrum of biosynthesized VNPs showing absorption bands.

The comprehensive physicochemical characterization confirmed the successful biosynthesis of crystalline VNPs using *P. aerogenes*. XRD analysis revealed sharp, well-defined reflections within the 2*θ* range of 10°–75° (Fig. 2A). The diffraction peaks indexed to the (200), (001), (101), (110), (011), (002), (102), (601), and (303) planes are consistent with the orthorhombic V_2_O_5_ phase, confirming the formation of crystalline vanadium pentoxide nanoparticles, consistent with prior reports of biologically synthesized vanadium oxide nanostructures.^32,33^ The broad amorphous background is attributed to residual biological organic material associated with bacterial capping agents.

FTIR analysis revealed distinct absorption bands corresponding to various functional groups (Fig. 2B). A broad absorption near 3435 cm^−1^ was observed, while peaks at 2924 cm^−1^ and 2854 cm^−1^ were detected. A prominent band at 1632 cm^−1^ was also present in the spectrum. Specifically, peaks at 1631.75 cm^−1^ (amide I, C=O stretching), 1457.01 cm^−1^ (C–H bending), and 1114.73 cm^−1^ (C–O stretching), alongside signals in the fingerprint region (739.23 and 535.09 cm^−1^), confirm the involvement of bacterial biomolecules such as proteins and polysaccharides as both reducing and stabilizing agents, consistent with the biomolecule-assisted capping mechanisms described.^34^

**Fig. 3:**
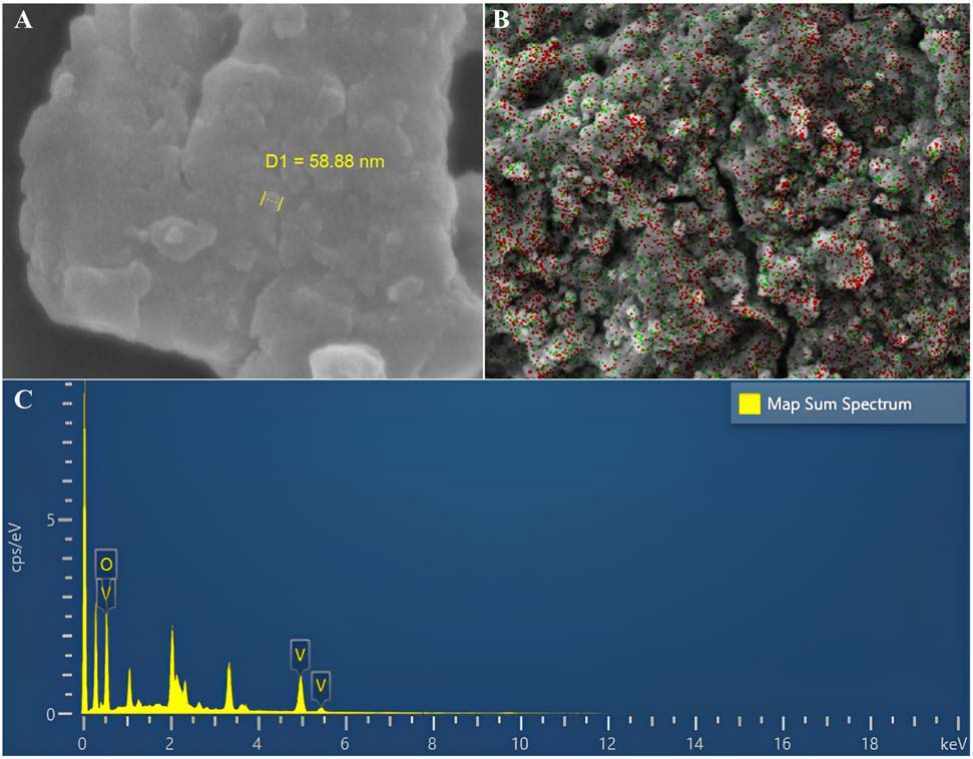
FESEM and EDX characterization of biosynthesized VNPs. (A) FESEM micrograph at 135k× magnification (scale bar = 200 nm) showing quasi-spherical particle morphology with a representative size measurement of 58.88 nm. (B) EDX elemental mapping overlay showing the spatial distribution of vanadium (V, green) and oxygen (O, red) across the sample surface (scale bar = 10 µm). (C) EDX spectrum confirming V and O as the sole detected elements; quantitative analysis yielded V (24.98 wt%, 9.47 at%) and O (75.02 wt%, 90.53 at%).

FESEM imaging revealed predominantly spherical to quasi-spherical VNPs with representative particle size measurements of 49.34 nm and 58.88 nm (average ∼ 54 nm), consistent with moderate dispersity and occasional minor agglomeration attributable to sample preparation (Fig. 3A). Nanoparticles within this size range are known to exhibit efficient cellular uptake and strong antibacterial interactions.^35,36^ The observed morphological uniformity underscores the reliability of biologically mediated synthesis compared with conventional chemical routes, which often yield heterogeneous morphologies and residual contaminants. EDX elemental mapping confirmed the uniform spatial distribution of vanadium and oxygen across the sample (Fig. 3B), with quantitative analysis yielding V (24.98 wt%, 9.47 at%) and O (75.02 wt%, 90.53 at%) as the sole detected elements (Fig. 3C), confirming phase purity and the effectiveness of the calcination and washing steps in eliminating residual biological material. The V : O atomic ratio is consistent with the orthorhombic V_2_O_5_ phase identified by XRD.

**Fig. 4:**
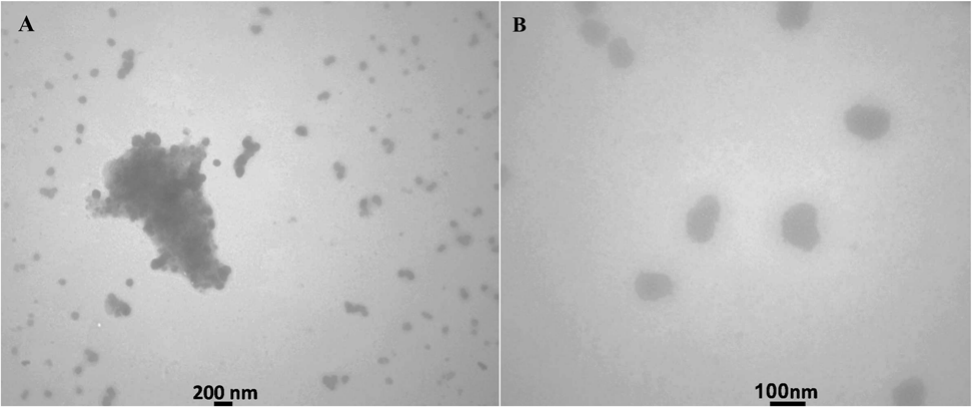
TEM micrographs of biosynthesized VNPs at (A) lower magnification (scale bar = 200 nm) showing overall particle dispersion, and (B) higher magnification (scale bar = 100 nm) confirming spherical to quasi-spherical morphology.

TEM micrographs confirmed the predominantly spherical to quasi-spherical morphology of the VNPs, with well-dispersed particles visible at both low (Fig. 4A) and high magnification (Fig. 4B), exhibiting sizes ranging approximately 20–80 nm, consistent with FESEM measurements. The relatively low electron contrast is attributed to the biological organic capping layer surrounding the vanadium oxide core, characteristic of biogenically synthesized nanoparticles.

**Fig. 5:**
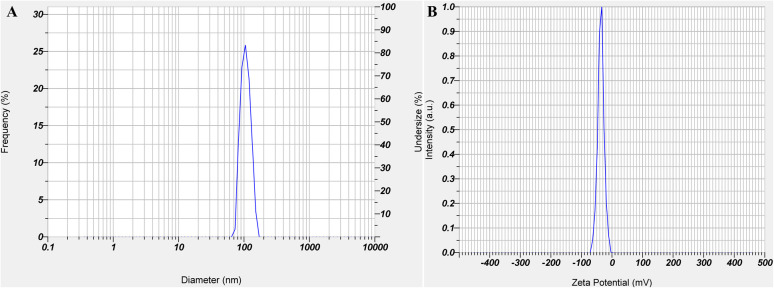
Colloidal characterization of biosynthesized VNPs. (A) DLS size distribution showing a modal hydrodynamic diameter of 98.5 nm (mean: 100.5 ± 0.4 nm, PDI: 0.663 ± 0.102, *n* = 3). (B) Zeta potential distribution showing a mean surface charge of −36.3 ± 1.35 mV (*n* = 3), indicative of moderate colloidal stability.

DLS analysis revealed a modal hydrodynamic diameter of 98.5 nm (mean: 100.5 ± 0.4 nm, PDI: 0.663 ± 0.102, *n* = 3; Fig. 5A), exceeding the dry particle dimensions observed by FESEM and TEM due to the hydration shell of surface-adsorbed biological capping molecules—a well-documented phenomenon for biogenic nanoparticles. The elevated PDI reflects the inherently polydisperse character of biological synthesis. Zeta potential measurements yielded a mean of −36.3 ± 1.35 mV (Fig. 5B), indicating moderate colloidal stability through electrostatic repulsion, consistent with surface-adsorbed negatively charged biomolecules identified by FTIR.

### 3.3 Piezocatalytic degradation of methylene blue

**Fig. 6:**
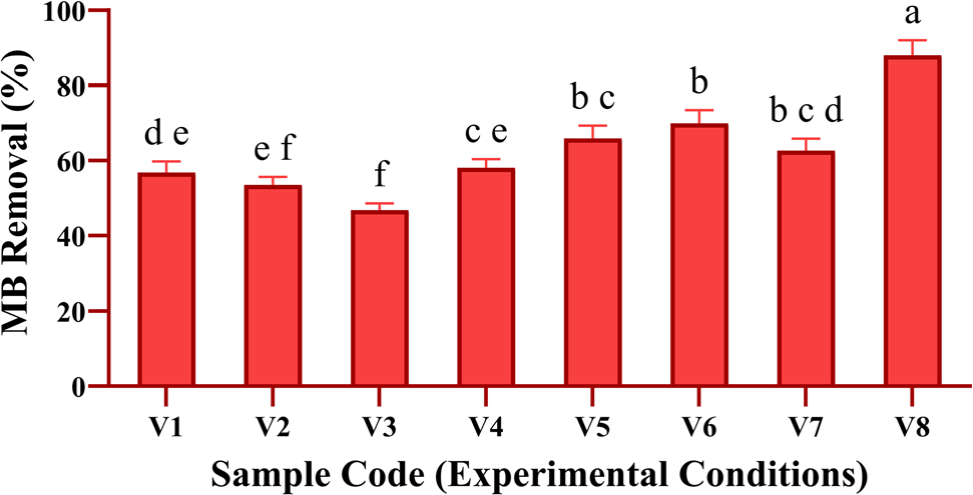
Piezocatalytic MB removal efficiency (%) of different biosynthesized vanadium catalyst formulations (V1–V8) under ultrasonication. V8 demonstrates the highest activity at 88.0%. Error bars represent standard deviation (*n* = 3); bars sharing no common letter differ significantly (one-way ANOVA with Tukey's *post hoc* test, *p* < 0.05).

Eight biosynthesized vanadium nanoparticle formulations (V1–V8) were evaluated for their piezocatalytic degradation efficiency of MB (Fig. 6). Distinct variations in degradation efficiency were observed among the formulations. V8 exhibited the highest piezocatalytic activity, achieving 88.0% MB removal. Optimization experiments demonstrated that both the cell-disruption method and calcination temperature profoundly influenced the structural and catalytic properties of VNPs. Among the tested formulations, the ethanol-lysed and calcined V8 sample exhibited the highest piezocatalytic performance. This superior activity is likely due to the ethanol-mediated extraction preserving key surface functional groups and preventing the denaturation of organic capping molecules, thereby maintaining electronic conductivity and enhancing active-site availability. The superior performance of samples calcined at 250 °C compared with 150 °C or non-calcined variants further confirms the importance of thermal treatment in improving crystallinity and charge-carrier mobility as reported in earlier research.^37,38^

The piezocatalytic activity of the synthesized VNPs aligns with documented piezocatalytic behaviors in vanadium-containing metal oxide frameworks, where nanoscale lattice distortions and surface oxygen vacancies effectively break local inversion symmetry.^39,40^ Ultrasonication-induced acoustic waves provide the mechanical stimulation necessary to activate this localized piezoelectric response, driving electron–hole pair generation and ROS formation that underpin the observed catalytic activity.^41,42^

**Fig. 7:**
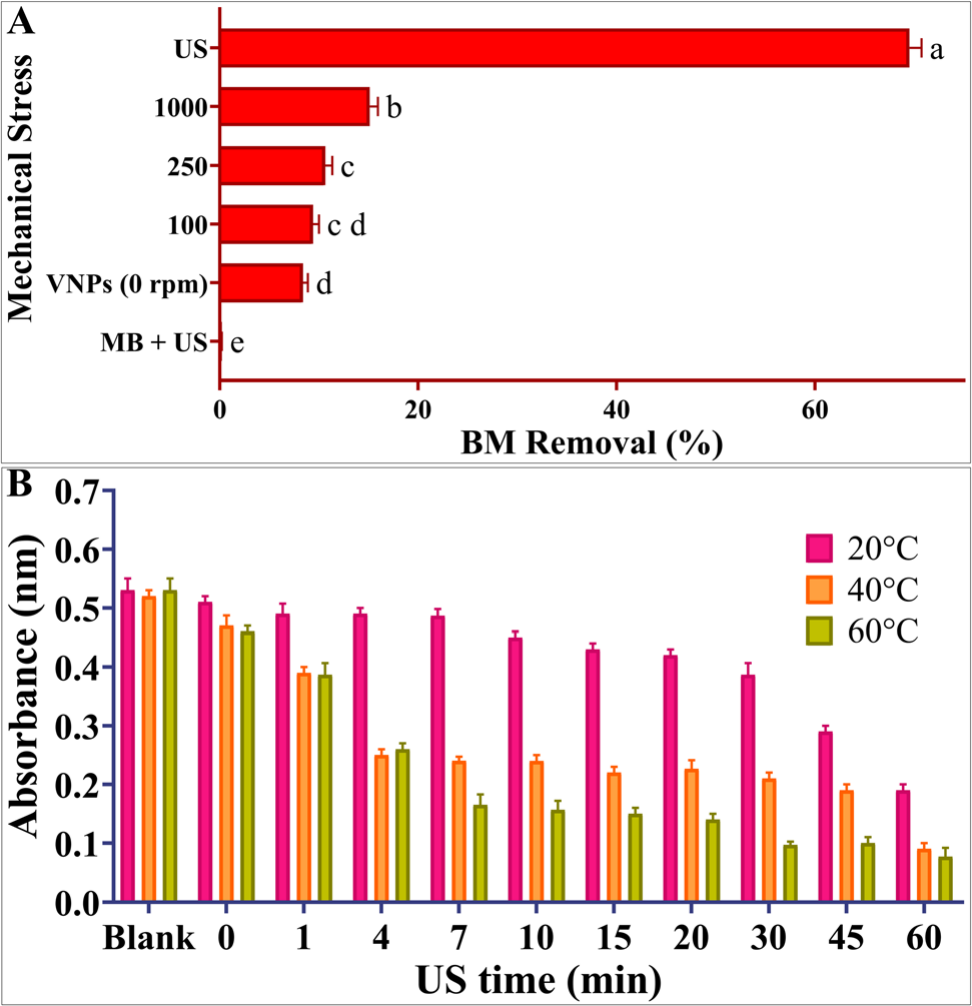
Effect of mechanical stress on MB degradation. (A) MB removal (%) under different conditions: MB + US control (no VNPs), VNPs (0 rpm) adsorption control, mechanical agitation (100, 250, 1000 rpm), and ultrasonication (US). Bars sharing no common letter differ significantly (one-way ANOVA with Tukey's *post hoc* test, *p* < 0.05; *n* = 3, error bars represent SD). (B) MB degradation kinetics at 20, 40, and 60 °C; 40 °C and 60 °C significantly outperform 20 °C at all time points from 0 min onward (two-way ANOVA with Dunnett's *post hoc* test, *p* < 0.001 to *p* < 0.0001; blank: ns; *n* = 3, error bars represent SD).

To confirm that MB removal was attributable to VNP-mediated piezocatalysis rather than spontaneous degradation, adsorption, or ultrasonication alone, appropriate controls were included in the mechanical activation experiment (Fig. 7A). The MB + US control, in which MB was subjected to ultrasonication in the absence of VNPs, showed negligible removal (∼0%), confirming that ultrasonication alone contributes negligibly to dye degradation. The VNPs (0 rpm) adsorption control resulted in approximately 8–9% MB removal, attributable to passive dye adsorption onto the VNP surface in the absence of mechanical activation. These controls collectively confirm that the observed MB removal is driven by VNP-mediated piezocatalytic activity rather than adsorption or acoustic effects alone.

Mechanical agitation incrementally increased MB degradation from ∼8–9% under static conditions to 15.1% at maximum agitation (1000 rpm), while ultrasonication produced substantially higher MB removal, achieving 69.5%. This marked improvement is attributed to acoustic cavitation, which generates localized high-energy microenvironments that promote charge separation and piezoelectric polarization, thereby facilitating electron–hole pair generation and ROS formation.^43^ Temperature-dependent ultrasonication tests (Fig. 7B) revealed significant differences in degradation kinetics. At 40 °C and 60 °C, substantially lower absorbance values were achieved compared to 20 °C at every time point from 0 min onward, with comparable degradation levels between 40 °C and 60 °C reached within 4 min, significantly accelerating reaction kinetics and confirming the thermal dependence of the piezocatalytic process (two-way ANOVA with Dunnett's *post hoc* test *vs.* 20 °C, *p* < 0.001 to *p* < 0.0001).

**Fig. 8:**
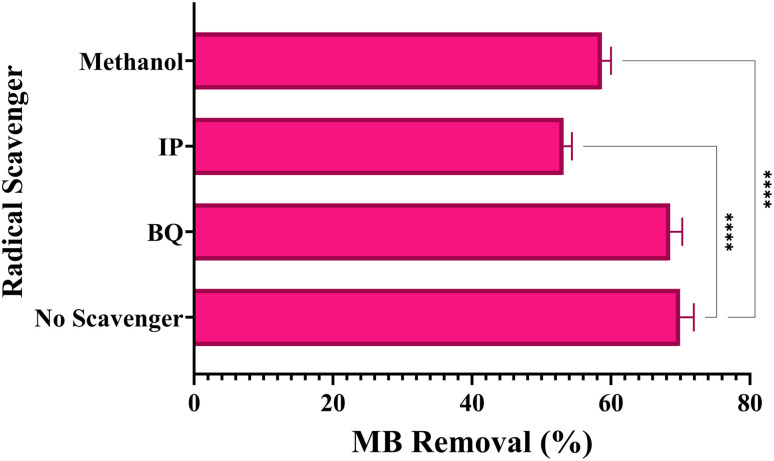
Effect of radical scavengers on piezocatalytic MB degradation by VNPs. Methanol and isopropanol (IP) significantly reduced MB removal compared to the no-scavenger control (****p < 0.0001 for both; one-way ANOVA with Dunnett's *post hoc* test), whereas benzoquinone (BQ) showed no significant effect (*p* = 0.588, ns). *n* = 3, error bars represent SD.

The role of ROS in piezocatalytic MB degradation was investigated using specific radical scavengers (Fig. 8). When BQ, a superoxide radical (˙O_2_^−^) scavenger, was introduced, degradation efficiency decreased slightly to 68.5% compared to 69.9% without scavengers, a difference that was not statistically significant. More significant inhibition was observed with hydroxyl radical (˙OH) scavengers: IP reduced MB degradation to 53.2%, while methanol decreased it to 58.7%. These results demonstrate that ˙OH, rather than ˙O_2_^−^, radicals are the primary oxidative species driving the degradation process. These results align with the mechanism where piezocatalytically generated holes oxidize water to form ˙OH radicals, which subsequently drive the oxidative decomposition of organic molecules.^44^

### 3.4 Piezocatalytic degradation kinetics

The kinetics of methylene blue (MB) degradation by the biosynthesized vanadium nanoparticles (VNPs) were investigated to evaluate the effect of temperature on the piezocatalytic degradation process. Degradation experiments were performed at 20, 40, and 60 °C under identical ultrasonic activation conditions (300 W). The experimental data were fitted to zero-order, pseudo-first-order, and second-order kinetic models. The pseudo-first-order model, derived from the Langmuir–Hinshelwood kinetic model, is widely used to describe the degradation kinetics of organic pollutants in heterogeneous photocatalytic and piezocatalytic systems.^45,46^ The kinetic equations are expressed as follows:

Zero-order model:*C*_*t*_ = *C*_0_ − *kt*

Pseudo-first-order model:
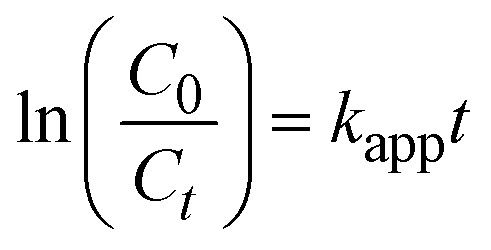


Second-order model:
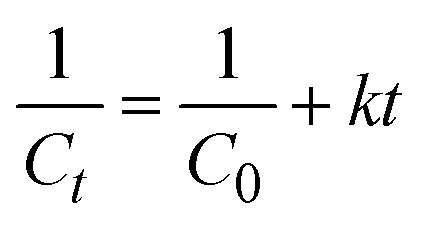
where *C*_0_ and *C*_*t*_ represent the initial and residual concentrations of methylene blue at reaction time *t*, respectively, and *k* or *k*_app_ denotes the apparent reaction rate constant. Since absorbance is directly proportional to concentration according to the Beer–Lambert law, the measured absorbance values were used to estimate the corresponding dye concentrations for kinetic analysis. The kinetic parameters and coefficients of determination (*R*^2^) for all three models at each temperature are summarized in Table 2.

**Table 2:** Kinetic parameters and coefficients of determination (*R*^2^) for the zero-order, pseudo-first-order, and second-order models describing the piezocatalytic degradation of methylene blue by biosynthesized vanadium nanoparticles (VNPs) at different temperatures

Model	Parameter	20 °C	40 °C	60 °C
Zero-order	*k*_0_ (AU min^−1^)	0.0529	0.0678	0.0829
	*R* ^2^	0.8166	0.8277	0.8472
Pseudo-first-order	*k*_app_ (min^−1^)	0.0775	0.1320	0.1933
	*R* ^2^	0.7091	0.8310	0.9485
Second-order	*k*_2_ (AU^−1^ min^−1^)	0.1225	0.3134	0.5664
	*R* ^2^	0.5931	0.6572	0.9471

**Fig. 9:**
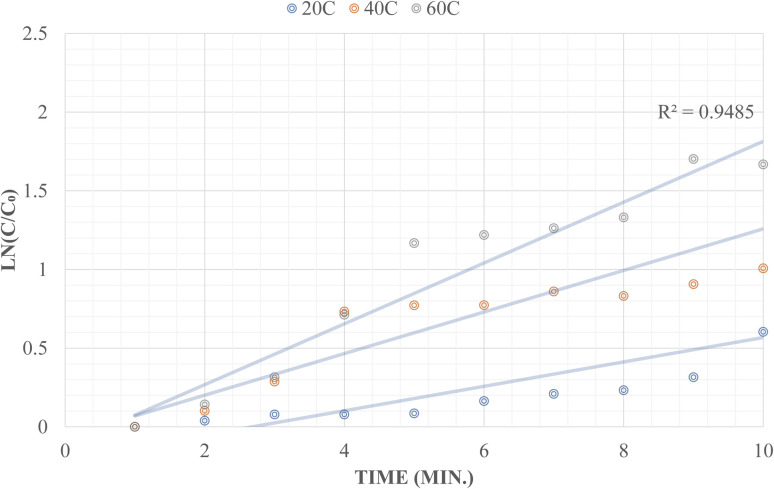
Pseudo-first-order kinetic plots for the piezocatalytic degradation of methylene blue by biosynthesized VNPs at 20, 40, and 60 °C under ultrasonic activation (300 W). Linear regression analysis was performed using the Langmuir–Hinshelwood pseudo-first-order kinetic model; coefficients of determination (*R*^2^) are shown for each temperature.

Comparison of the regression coefficients demonstrated that degradation behaviour was strongly influenced by reaction temperature (Fig. 9). At 20 °C, the zero-order model showed the highest correlation coefficient (*R*^2^ = 0.8166), whereas the pseudo-first-order and second-order models exhibited lower goodness of fit (*R*^2^ = 0.7091 and 0.5931, respectively). At 40 °C, the zero-order and pseudo-first-order models produced comparable correlations (*R*^2^ = 0.8277 and 0.8310, respectively), indicating a gradual transition in degradation kinetics. In contrast, at 60 °C, the pseudo-first-order model provided the best fit (*R*^2^ = 0.9485), confirming that the degradation process at the optimal temperature is best described by the pseudo-first-order kinetic model. The apparent rate constant (*k*_app_) increased progressively from 0.0775 min^−1^ at 20 °C to 0.1320 min^−1^ at 40 °C and 0.1933 min^−1^ at 60 °C, further confirming that elevated temperature accelerated the degradation process. The enhanced kinetic performance at higher temperatures is consistent with improved mass transfer and increased ROS generation during ultrasonic activation, thereby promoting the oxidative degradation of MB.^47,48^ These findings suggest that elevated temperature acts synergistically with ultrasonic activation to improve the piezocatalytic efficiency of the biosynthesized VNPs.

### 3.5 Antimicrobial and antibiofilm activity

The antimicrobial activity of VNPs was assessed against MDR *S. aureus* and XDR *P. aeruginosa* with and without ultrasound exposure (Table 3). Without sonication, inhibition zones measuring approximately 7.13 ± 0.65 mm and 7.20 ± 0.40 mm were recorded for *S. aureus* and *P. aeruginosa*, respectively. Following ultrasonication, inhibition zones increased significantly to 13.83 ± 0.80 mm for *S. aureus* and 8.80 ± 0.55 mm for *P. aeruginosa*. Ultrasonicated VNPs displayed superior bactericidal activity compared with non-sonicated samples, possibly attributable to enhanced particle dispersion and increased surface area exposure under acoustic conditions, although direct piezoelectric contribution cannot be excluded and warrants further investigation. Importantly, sonicated VNPs produced inhibition zones statistically below those of azithromycin (15.77 ± 0.80 mm for *S. aureus*, *p* = 0.0017; 11.23 ± 0.65 mm for *P. aeruginosa*, *p* = 0.0003), indicating that while piezocatalytically activated VNPs demonstrate promising antibacterial activity, their efficacy does not yet match that of the conventional antibiotic standard under the tested conditions.

**Table 3:** Antimicrobial activity of VNPs against MDR *S. aureus* and XDR *P. aeruginosa* with and without ultrasound exposure^a^

Treatment condition	Inhibition zone (mm) *S. aureus*	Inhibition zone (mm) XDR *P. aeruginosa*
VNPs (without ultrasound)	7.13 ± 0.65^a^	7.20 ± 0.40^a^
VNPs (with ultrasound)	13.83 ± 0.80^b^	8.80 ± 0.55^b^
Azithromycin (control)	15.77 ± 0.80^c^	11.23 ± 0.65^c^

a Values sharing no common superscript letter differ significantly within each strain (two-way ANOVA with Tukey's *post hoc* test; *n* = 3, mean ± SD).

**Fig. 10:**
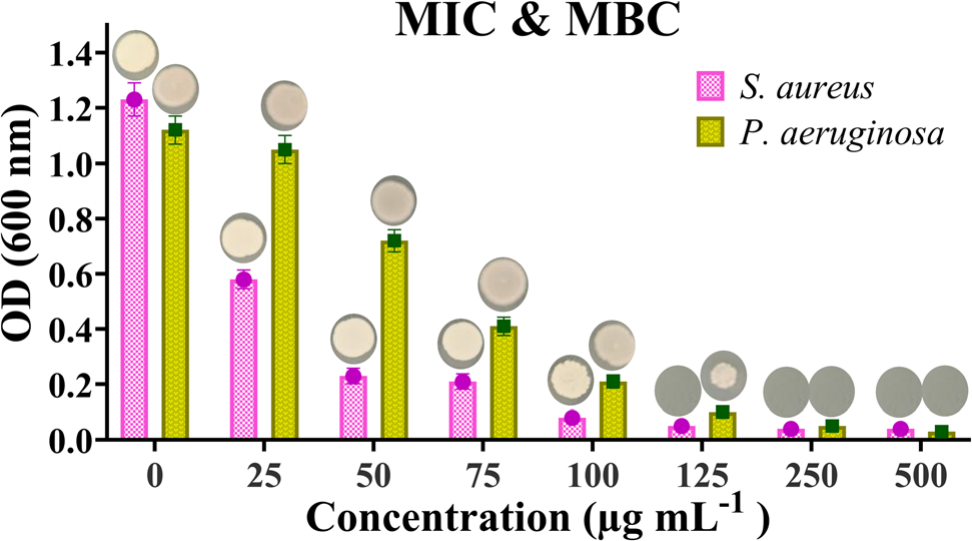
Antimicrobial activity of sonicated VNPs. Concentration-dependent inhibition of MDR *S. aureus* and XDR *P. aeruginosa* by OD_600_ measurements, with insets showing corresponding spot assays that confirm the growth suppression. Two-way ANOVA with Dunnett's *post hoc* test confirmed significant growth inhibition at all concentrations *vs.* untreated control (*p* < 0.0001), except 25 µg mL^−1^ for *P. aeruginosa* (*p* < 0.05). *n* = 3, error bars represent SD.

Biosynthesized and ultrasonicated VNPs demonstrated significant concentration-dependent antimicrobial activity against both clinical isolates (Fig. 10). For MDR *S. aureus*, growth inhibition became prominent at 75–100 µg mL^−1^, while XDR *P. aeruginosa* required higher concentrations (100–125 µg mL^−1^) to achieve comparable inhibition. Complete bactericidal effects were observed at 125 µg mL^−1^ for *S. aureus* and 250 µg mL^−1^ for *P. aeruginosa*.

The observed differences in sensitivity between MDR *S. aureus* and XDR *P. aeruginosa* strains reflect fundamental disparities in their cell envelope architecture and resistance determinants.^49,50^ The higher MBC value for *P. aeruginosa* (250 µg mL^−1^) compared with *S. aureus* (125 µg mL^−1^) is consistent with its advanced defense systems, including the MexAB-OprM efflux pumps, robust biofilm formation, and protective lipopolysaccharide-rich outer membrane.^51,52^ The mechanism of VNP antibacterial action is likely multifactorial, involving cell membrane disruption, oxidative stress *via* ROS generation, inhibition of respiratory enzymes, and interference with DNA replication.^53^

**Fig. 11:**
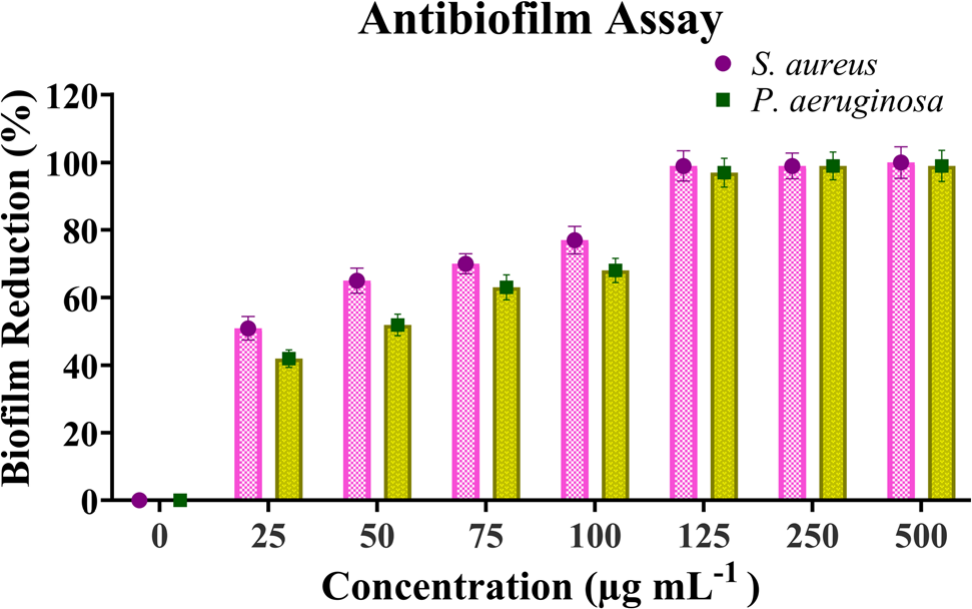
Antibiofilm activity of VNPs against resistant bacterial strains. Concentration-dependent biofilm reduction in MDR *S. aureus* and XDR *P. aeruginosa* measured by crystal violet assay. Two-way ANOVA with Dunnett's *post hoc* test confirmed significant biofilm inhibition at all tested concentrations *vs.* untreated control (*p* < 0.0001). *n* = 3, error bars represent SD.

VNPs demonstrated concentration-dependent inhibition of biofilm formation for both bacterial strains (Fig. 11). *S. aureus* exhibited greater susceptibility to antibiofilm effects compared to *P. aeruginosa* across all tested concentrations. Complete inhibition (>95%) was achieved at 125 µg mL^−1^ for both pathogens. This effect is particularly significant given that biofilm-associated infections account for nearly 80% of chronic bacterial infections and exhibit up to 1000-fold increased resistance to antibiotics.^54^ The slightly greater antibiofilm activity against *S. aureus* compared to *P. aeruginosa* likely arises from differences in extracellular polymeric substance composition and quorum sensing regulation.^55^ The ability of VNPs to inhibit biofilm establishment at sub-bactericidal concentrations suggests a dual mechanism involving interference with adhesion and signaling pathways.^56^

Collectively, the results position biologically derived VNPs as a promising multifunctional platform, pending validation in physiologically relevant models.

## 4. Conclusions

This study demonstrates, to the best of our knowledge, the first biogenic synthesis of vanadium nanoparticles using *P. aerogenes*, offering a non-toxic and scalable alternative to conventional chemical methods. The optimized VNPs exhibited dual functionality, serving as highly efficient piezocatalysts for organic pollutant degradation and potent antimicrobial agents against MDR pathogens. The enhanced activity observed under ultrasonication is consistent with acoustic-driven mechanisms, including improved particle dispersion and potential piezoelectric activation, though direct confirmation through piezoelectric coefficient measurements (*e.g.*, *d*_33_) remains an important direction for future work. These findings present a versatile, eco-friendly platform for addressing water pollution and antimicrobial resistance, with translational applicability subject to further *in vivo* and toxicological validation.

## Author contributions

Karzan Qurbani: conceptualization, formal analysis, methodology, supervision, writing – review and editing; Safin Hussein: formal analysis, supervision, writing – original draft; Bahra Abbas Ibrahim: formal analysis, methodology, writing – review and editing; Hedi Muhammadamin Khalid: formal analysis, methodology, writing – review and editing; Doaa K. Al-seleet: software, writing – review and editing; Abdulmalik Fareeq Saber: software, validation, writing – review and editing; Vania Hassan Muhammed: formal analysis, methodology, writing – review and editing; Shnan Abdulrahman Hassan: formal analysis, methodology, writing – review and editing; Saman M. Mohammed: formal analysis, methodology, validation, writing – review and editing.

## Conflicts of interest

There are no conflicts to declare.

## Data Availability

The data supporting this article have been included within the manuscript.

## References

[cit1] Douglas E. J. A., Wulandari S. W., Lovell S. D., Laabei M. (2023). Microb. Biotechnol..

[cit2] Ahmed S. K., Hussein S., Qurbani K., Ibrahim R. H., Fareeq A., Mahmood K. A., Mohamed M. G. (2024). J. Med. Surg. Public Health.

[cit3] Punz B., Christ C., Waldl A., Li S., Liu Y., Johnson L., Auer V., Cardozo O., Farias P. M. A., Andrade A. C. D. S., Stingl A., Wang G., Li Y., Himly M. (2025). Environ. Sci.: Nano.

[cit4] Hussein S., Sulaiman S., Ali S., Pirot R., Qurbani K., Hamzah H., Hassan O., Ismail T., Ahmed S. K., Azizi Z. (2023). Biol. Trace Elem. Res..

[cit5] Hachem K., Ansari M. J., Saleh R. O., Kzar H. H., Al-Gazally M. E., Altimari U. S., Hussein S. A., Mohammed H. T., Hammid A. T., Kianfar E. (2022). BioNanoScience.

[cit6] Krishnia L. , Thakur P. and Thakur A., Synthesis and Applications of Nanoparticles, 2022, pp. 45–59

[cit7] Barawi S. S., Qurbani K. A., Ali S. M., Hussein S. H., Aziz D. M., Hamasalih R. O., Ahmed R. B., Hamzah H. M. (2024). Biologia.

[cit8] Bokolia M., Baliyan D., Kumar A., Das R., Kumar R., Singh B. (2025). Int. J. Environ. Anal. Chem..

[cit9] Qurbani K., Hamzah H. (2020). Arch. Microbiol..

[cit10] Della-Flora I. K., de Andrade C. J. (2023). Nanoscale.

[cit11] Efunnuga A., Efunnuga A., Onivefu A. P., Ifijen I. H., Maliki M., Omorogbe S. O., Olugbemide A. D. (2024). BioNanoScience.

[cit12] Bansal S., Singh A., Poddar D., Thakur S., Jain P. (2024). Prep. Biochem. Biotechnol..

[cit13] Zhang L., Yang Z., Zeng W., Liang B., Huang Z., Wang Q., Li Z., Chen T., Yan B. (2025). ACS ES&T Eng..

[cit14] Ibrahim W. M., Amiri O., Ahmed S. S., Muhammed H. Y., Mahmood P. H., Qurbani K. A., Abdulrahman N. A., Younis K. A., Omer P. K. (2024). Results Eng..

[cit15] Amiri O., Qurbani K. A., Babakr K. A., Omer P. K., Guo L. J., Najmuldeen H. H. R., Bertau M., Mahmood P. H., Ahmed S. S., Jamal M. A. (2025). Adv. NanoBiomed Res..

[cit16] Meng F., Guo C., Cui T., Xu M., Chen X., Xu H., Liu C., Chen S. (2025). Mater. Chem. Front..

[cit17] Basu R., Mangamma G., Dhara S. (2022). ACS Omega.

[cit18] Qurbani K., Wsw H., Khdhr R., Hussein S., Ibrahim B., Mahmood A., Hama L., Ibrahim F., Amiri O. (2025). Sci. Rep..

[cit19] Padwal Y., Chauhan R., Chaudhary I. J., Late D. J., Ashokkumar M., Gosavi S. (2025). Energy Adv..

[cit20] Kumar M., Vaish R., Elqahtani Z. M., Kebaili I., Al-Buriahi M. S., Sung T. H., Hwang W., Kumar A. (2022). J. Mater. Res. Technol..

[cit21] Ko Y., Kang S., Yang Y., Lee J., Hur H. G. (2025). J. Microbiol. Biotechnol..

[cit22] Marucci M., Tangerina P., Abdelhamid A. G., Yousef A. E. (2023). Antibiotics.

[cit23] Pagnucco G., Overfield D., Chamlee Y., Shuler C., Kassem A., Opara S., Najaf H., Abbas L., Coutinho O., Fortuna A., Sulaiman F., Farinas J., Schittenhelm R., Catalfano B., Li X., Tiquia-Arashiro S. M. (2023). Front. Microbiol..

[cit24] Kadeřábková N., Mahmood A. J. S., Mavridou D. A. I. (2024). npj Antimicrob. Resist..

[cit25] Prakruthi R., Deepakumari H. N., Revanasiddappa H. D., Alfaisal F. M., Alam S., Majdi H. S., Amir khan M., Ukkund S. J. (2024). AIP Adv..

[cit26] Ali A. T., Abdul Karem L. K. (2024). Baghdad Sci. J..

[cit27] Bin Chan Y., Selvanathan V., Tey L. H., Akhtaruzzaman M., Anur F. H., Djearamane S., Watanabe A., Aminuzzaman M. (2022). Nanomaterials.

[cit28] Qurbani K., Amiri O., Hamzah H. (2026). Sci. Rep..

[cit29] Hao L., Wang X., Shi J., Li L., Hao X. (2023). Front. Environ. Sci..

[cit30] Adamiak J. W., Ajmal L., Zgurskaya H. I. (2024). J. Bacteriol..

[cit31] Balta I., Lemon J., Gadaj A., Cretescu I., Stef D., Pet I., Stef L., McCleery D., Douglas A., Corcionivoschi N. (2025). Front. Microbiol..

[cit32] Hu P., Hu P., Vu T. D., Li M., Wang S., Ke Y., Zeng X., Mai L., Long Y. (2023). Chem. Rev..

[cit33] Hieu N. H., Vinh An T. T., Thu N. M., Son N. H., Hoang Yen L. D., Dat N. M., Hoai Nam N. T., Do Dat T., Cong Minh D. T., Hanh N. T., Ngoc Hieu N. T. (2024). Biochem. Biophys. Res. Commun..

[cit34] Carrapiço A., Martins M. R., Caldeira A. T., Mirão J., Dias L. (2023). Microorganisms.

[cit35] Zhang W., Taheri-Ledari R., Ganjali F., Mirmohammadi S. S., Qazi F. S., Saeidirad M., KashtiAray A., Zarei-Shokat S., Tian Y., Maleki A. (2022). RSC Adv..

[cit36] Rajamohan R., Thamaraiselvi K., Raorane C. J., Murugavel K., Govindasamy C., Kim S.-C., Sun S. (2025). Bioengineering.

[cit37] Gavhane D. S., Sontakke A. D., van Huis M. A. (2023). ACS Appl. Nano Mater..

[cit38] Nzengu P. D., Hlongwa N. W., Sekhosana K. E., Kebede M. A. (2025). RSC Adv..

[cit39] Kumar M., Vaish R., Kebaili I., Boukhris I., Kwang Benno Park H., Hwan Joo Y., Hyun Sung T., Kumar A. (2023). Sci. Rep..

[cit40] Ding L., Zhu Y., Zhang Q., Li Y., Hou J., Xiao Z. (2026). Chem. Synth..

[cit41] Porwal C., Verma S., Kumar M., Gaur A., Chauhan V. S., Vaish R., Kebaili I., Boukhris I., Park H. K. B., Joo Y. H., Sung T. H., Kumar A. (2023). Sci. Rep..

[cit42] Guo J., Chen F., Wei Y., Sun S. (2026). Chem. Commun..

[cit43] Jajko-Liberka G., Anagha M. G., Chytrosz-Wróbel P., Kubisiak P., Kulig W., Cwiklik L., Kotarba A. (2025). Ultrason. Sonochem..

[cit44] Henrique P., Nunes H., Marinho J. Z., Nascimento L. L., Ricardo I. A., Montoro L. A., Camillo L. K. S., Krambrock K., Wang C., Otavio A., Patrocinio T. (2025). ACS Appl. Eng. Mater..

[cit45] Chong M. N., Jin B., Chow C. W. K., Saint C. (2010). Water Res..

[cit46] Herrmann J. M. (1999). Catal. Today.

[cit47] Wang K., Han C., Li J., Qiu J., Sunarso J., Liu S. (2022). Angew. Chem., Int. Ed..

[cit48] Liu J., Qi W., Xu M., Thomas T., Liu S., Yang M. (2023). Angew. Chem., Int. Ed..

[cit49] Bharadwaj A., Rastogi A., Pandey S., Gupta S., Sohal J. S. (2022). BioMed Res. Int..

[cit50] Birlutiu V., Birlutiu R. M. (2025). Microorganisms.

[cit51] Mazza L., Bory A., Luscher A., Kloehn J., Wolfender J. L., van Delden C., Köhler T. (2024). Front. Microbiol..

[cit52] Tabarzad M., Torshabi M., Haeri A., Fathi F., Mortazavi S. M. (2026). Bioorg. Med. Chem..

[cit53] Subramanian P., David S. A. (2024). Uttar Pradesh J. Zool..

[cit54] Sahoo J., Sarkhel S., Mukherjee N., Jaiswal A. (2022). ACS Omega.

[cit55] Sharma S., Mohler J., Mahajan S. D., Schwartz S. A., Bruggemann L., Aalinkeel R. (2023). Microorganisms.

[cit56] Carvalho-Silva J. M., Teixeira A. B. V., de Cássia Oliveira V., de Carvalho A. C. W., Ferreira-Duarte M. P., de Freitas O., Schiavon M. A., dos Reis A. C. (2024). J. Drug Delivery Sci. Technol..

